# Middle meningeal artery embolization for chronic subdural hematoma: a systematic review

**DOI:** 10.3389/fneur.2023.1259647

**Published:** 2023-10-10

**Authors:** Yoshihiro Omura, Taichi Ishiguro

**Affiliations:** Department of Neurosurgery, Tokyo Women's Medical University Yachiyo Medical Center, Yachiyo, Chiba, Japan

**Keywords:** embolization, chronic subdural hematoma, middle meningeal artery, recurrence, endovascular

## Abstract

**Background:**

Chronic subdural hematoma (cSDH) is one of the most common diseases in neurosurgery. Middle meningeal artery embolization (MMAE) is reportedly an option to prevent recurrence or avoid surgery in patients with cSDH. This study was performed to review the evidence on MMAE for cSDH and evaluate its safety, efficacy, indications, and feasibility.

**Methods:**

We systematically reviewed the literature according to the PRISMA guidelines using an electronic database. The search yielded 43 articles involving 2,783 patients who underwent MMAE.

**Results:**

The hematoma resolution, recurrence, and retreatment rates in the MMAE-alone treatment group (*n* = 815) were 86.7%, 6.3%, and 9.6%, respectively, whereas those in the prophylactic MMAE with combined surgery group (*n* = 370) were 95.6%, 4.4%, and 3.4%, respectively. The overall MMAE-related complication rate was 2.3%.

**Conclusion:**

This study shows that MMAE alone is, although not immediate, as effective as evacuation surgery alone in reducing hematoma. The study also shows that combined treatment has a lower recurrence rate than evacuation surgery alone. Because MMAE is a safe procedure, it should be considered for patients with cSDH, especially those with a high risk of recurrence.

## Introduction

Chronic subdural hematoma (cSDH) is a common disease with an incidence of up to 58.1 per 100,000 person-years in patients aged > 65 years (1). cSDH is commonly treated by surgical evacuation through burr hole(s) to relieve the symptom caused by the mass effect of the hematoma. However, the recurrence rate ranges from 10% to 20% (2, 3). The use of antiplatelet drugs or anticoagulants, multiple recurrences, and advanced age are known risk factors for cSDH recurrence (4, 5).

The pathophysiology of cSDH involves the formation of neomembranes with fragile neovascularization, perfused mainly by distal branches of the middle meningeal artery (MMA) that have formed by inflammatory remodeling of the dura matter (6, 7). Therefore, endovascular MMA embolization (MMAE) has recently emerged as an alternative or adjunct modality to conventional surgical treatment to prevent the recurrence of cSDH.

This study was performed to review all published cases of MMAE for cSDH and assess the safety, efficacy, and indications of the procedure.

## Methods

### Study design

This systematic review follows the Preferred Reporting Items for Systematic Reviews and Meta-Analyses (PRISMA) guidelines (8).

### Literature search

An electronic literature search of the PubMed database was performed on 28 May 2023 using the following key terms: chronic subdural hematoma, meningeal artery, and embolization. All articles published from January 1976 to May 2023 were identified. Two reviewers (Y.O. and T.I.) independently screened the articles based on their title and abstract. One reviewer (Y.O.) then reviewed the full text of all relevant articles in detail to further assess the eligibility of the studies.

### Inclusion and exclusion criteria

The following inclusion and exclusion criteria were used for the systematic review.

#### Inclusion criteria

- Original research involving more than five cases of MMAE published in any peer-reviewed journal.
- English language.
- Sufficient post-embolization outcome data (at least one post-MMAE clinical and/or radiological follow-up and reporting of the rescue surgical treatment rate).
- Adult patients (>18 years old) treated for the first time by MMAE for cSDH.

#### Exclusion criteria

- Review articles, meta-analyses, comments, letters, and editorials.
- cSDH due to a vascular malformation (e.g., dural arteriovenous fistula, arteriovenous malformation) or intracranial tumor, or the presence of intracranial hypotension.
- Studies from the same author with duplicate patients.

### Data extraction and analysis

Data extraction was performed by one reviewer (Y.O.) using a predefined data extraction form. The following data were collected and analyzed: study design, sample population and size, patients’ baseline characteristics, antithrombotic therapy use, management strategy (including surgical and endovascular treatment), endovascular treatment success, outcome, follow-up duration, complications, cSDH recurrence rate, and the need for subsequent surgery.

Several definitions of resolution after MMAE were used among the studies, including complete resolution, near complete resolution (reduction of ≥90%), suboptimal resolution (reduction of ≥50%), and partial reduction, the latter two of which were ambiguous. Among these definitions, there were further differences in measurement methods, with some researchers using the hematoma volume for measurement and others using the hematoma thickness. In this study, we focused on the necessity of rescue surgery and defined resolution as radiological improvement.

Some reports included in this review defined recurrent cSDH as only symptomatic re-accumulation after surgical intervention, while others defined recurrent cSDH as also asymptomatic re-accumulation after surgical intervention. Both were included in recurrent cSDH in this review.

In MMAE for cSDH treatment, there are differences in the purpose of treatment and the characteristics of patients between MMAE as a sole treatment and MMAE as prophylaxis, in which surgery is performed before and after MMAE. In this study, we compared the data between reports in which all patients were treated with MMAE alone and reports in which all patients were treated with MMAE combined with surgery.

MMAE treatment success was defined as the successful embolization of the target vessel. All patients who failed to achieve embolization, including MMAE abort, were considered treatment failures. Complications of treatment were counted after excluding those considered to be complications related to surgery (evacuation), those with unknown details, and those with an unknown relationship to MMAE.

## Results

### Study characteristics

**Figure 1:**
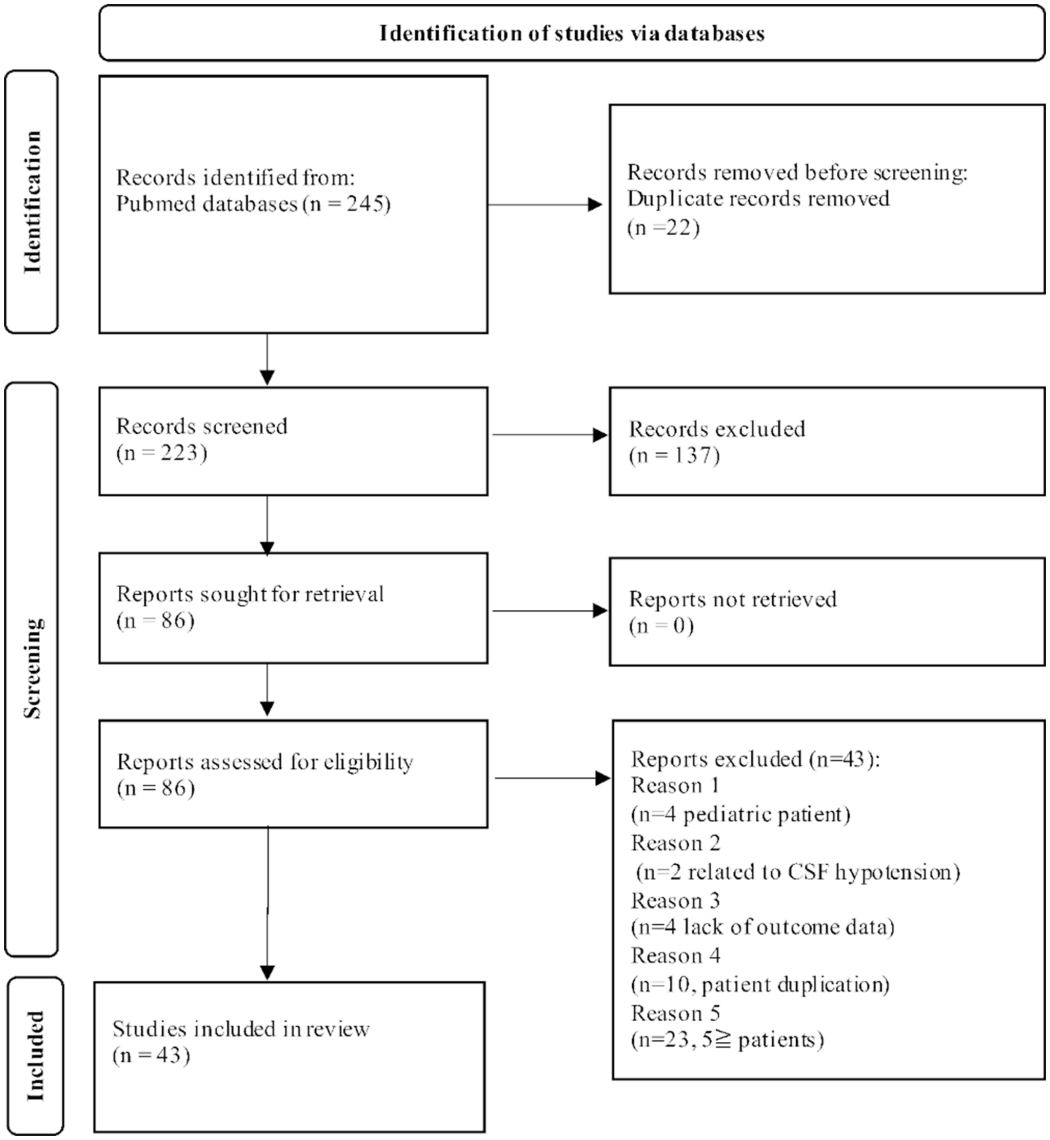
PRISMA flow chart of our systematic review. CSF, cerebrospinal fluid.

Our search strategy yielded 245 articles. Of these, 202 articles were excluded based on the exclusion criteria of the present review. A flow diagram of this study shown in Figure 1.

Forty-three articles were included in the final analysis, including 2 prospective uncontrolled studies, 1 prospective randomized study, and 40 retrospective studies involving 7 to 530 patients per series.

### Patient demographics and clinical and radiographic characteristics

A total of 2,783 patients underwent MMAE in the selected studies (Table 1). Their mean age was 71.2 years, and 71.1% were male. The available data showed that 90.6% of patients had symptomatic cSDH and that 19.9% had bilateral cSDH. Concurrent antiplatelet or anticoagulant use at the time of cSDH treatment was reported in 50.3% of patients.

**Table 1:** Studies and patient characteristics in this review.

Study	Year	Study design	No of patients	No of embolizations	Age (mean)	Male (%)	Unilateral/Bilateral	Symptomatic cSDH (%)	Antithrombotic therapy (%)
Kim et al. (5)	2017	Retrospective	20	20^*^	73.7	14 (70.0)	14/6	20 (100)	9 (45.0)
Ban et al. (9)	2018	Retrospective	72	72^*^	69.5	48 (66.7)	53/19	45 (62.5)	29 (40.3)
Waqas et al. (10)	2019	Retrospective	8	8	63.6	5 (62.5)	7/1	8 (100)	8 (100)
Saitoh et al. (11)	2019	Retrospective	8	8^*^	79	8 (100)	6/2	NR	2 (25.0)
Okuma et al. (4)	2019	Retrospective	17	17^*^	76.4	12 (70.6)	13/4	17 (100)	11 (64.7)
Nakagawa et al. (12)	2019	Retrospective	20	20^*^	78.3	14 (70)	12/8	20 (100)	4 (20.0)
Link et al. (13)	2019	Retrospective	49	60	69	32 (65.3)	38/11	NR	39 (77.5)
Yajima et al. (14)	2020	Retrospective	18	18^*^	78.5	16 (88.9)	15/3	NR	3 (16.7)
Shotar et al. (15)	2020	Retrospective	89	89^*^	74	68 (76.4)	74/15	89 (100)	71 (79.7)
Rajah et al. (16)	2020	Prospective	46	46^*^	71.7	31 (67.3)	40/6	44 (100)	14 (31.8)
Ng et al. (17)	2020	Prospective randomized	19	22	77.4	10 (52.9)	16/3	19 (100)	7 (31.8)
Mureb et al. (18)	2020	Retrospective	8	8	75.4	7 (87.5)	8/0	NR	NR
Joyce et al. (19)	2020	Retrospective	121	151	77.5	99 (81.8)	91/30	NR	66 (54.3)
Fan et al. (20)	2020	Retrospective	7	7	NR	NR	7/0	7 (100)	NR
Wei et al. (21)	2021	Retrospective	10	20	63.3	10 (100)	0/10	10 (100)	NR
Tiwari et al. (22)	2021	Retrospective	10	13	71.4	NR	7/3	10 (100)	6 (60.0)
Tanoue et al. (23)	2021	Retrospective	15	15	78	10 (66.7)	15/0	15 (100)	1 (6.6)
Schwarz et al. (24)	2021	Retrospective	41	44	73.3	33 (75.0)	38/3	44 (100)	19 (43.1)
Petrov et al. (25)	2021	Retrospective	10	15	66	7 (70.0)	5/5	10 (100)	0 (0)
Lee et al. (26)	2021	Retrospective	22	31	63.9	16 (72.7)	13/9	31 (100)	4 (18.2)
Kan et al. (27)	2021	Prospective	138	138^*^	69.8	98 (71.0)	122/16	NR	72 (52.2)
Gomez-Paz et al. (28)	2021	Retrospective	23	27	74	10 (43.5)	19/4	20 (87.0)	13 (56.5)
scovile et al. (29)	2022	Retrospective	208	208	NR	115 (55.3)	NR	NR	106 (52.0)
Saway et al. (30)	2022	Retrospective	100	100^*^	73	68 (68.0)	64/32	97 (97)	66 (66.0)
Samarage et al. (31)	2022	retrospective	37	37^*^	76.9	25 (67.6)	23/15	26 (70)	17 (45.9)
Salie et al. (32)	2022	Retrospective	52	52	74.2	38 (73.1)	52/0	47 (90.4)	37 (71.2)
Onyinzo et al. (33)	2022	Retrospective	50	50	79.6	42 (84.0)	50/0	NR	35 (70.0)
Mir et al. (34)	2022	Retrospective	56	56^*^	73	43 (76.8)	51/5	NR	23 (41.1)
Magidi et al. (35)	2022	Retrospective	61	61^*^	62.5	48 (78.7)	39/22	NR	34 (55.8)
Khorasanizadeh et al. (36)	2022	Retrospective	78	94	72	50 (64.1)	62/32	65 (83.3)	52 (66,7)
Housley et al. (37)	2022	Retrospective	44	48	73.3	25 (56.8)	40/4	NR	21 (47.7)
Fuentes et al. (38)	2022	Retrospective	322	322	NR	228 (70.8)	NR	NR	58 (18.0)
Enriquiz-Marulanda et al. (39)	2022	Retrospective	36	45	76	28 (62.2)	27/9	43 (95.6)	34 (75.5)
Dofuku et al. (40)	2022	Retrospective	9	9	85	6 (66.7)	9/0	9 (100)	5 (55.5)
Catapano et al. (41)	2022	Retrospective	66	84	70	51 (77.3)	48/18	50 (75.8)	32 (48.5)
Carpenter et al. (42)	2022	Retrospective	23	23^*^	80	15 (65.2)	13/10	NR	20 (87.0)
Wali et al. (43)	2023	Retrospective	8	8	80.5	6 (75.0)	3/5	NR	NR
Shehabeidin et al. (44)	2023	Retrospective	97	97	78	71 (73.2)	NR	NR	50 (51.5)
Seok et al. (45)	2023	Retrospective	9	13	77.3	8 (88.8)	13/0	13 (100)	7 (77.7)
Salem et al. (46)	2023	Retrospective	530	636	71.9	38 6(72.8)	424/106	NR	281 (53.0)
Martinez-Gutierrez et al. (47)	2023	Retrospective	57	66	66	45 (78.9)	48/9	NR	28 (49.5)
Liu et al. (48)	2023	Retrospective	53	53	68.1	42 (79.2)	53/0	NR	20 (38.7)
Krothapalli et al. (49)	2023	Retrospective	116	116	NR	80 (68.9)	116/0	NR	80 (69.0)
			2,783	3,027	71.2	1,968 (71.1)	1,748/435	759 (90.6)	1,384 (50.3)

cSDH, chronic subdural hematoma; NR, not reported. ^*^Treatment of bilateral cSDH counted as one treatment.

### Characteristics and outcomes of MMAE for cSDH

In total, 3,027 MMAE procedures were performed on 2,783 patients. The MMAE treatment success rate was 98.8%. In principle, treatment for bilateral cSDH was considered two embolization procedures, but some studies counted it as one treatment. This is clearly indicated in Table 2.

**Table 2:** Details and outcomes of MMAE.

Study	No of patients	No of embolizations	Mean follow-up	Embolization material (*n*)	MMAE alone (%)	Prophylactic MMAE (%)	Upfront-MMAE (%)	Recurrent cSDH (%)	Complications (%)	Resolution (%)	Recurrence (%)	Rescue surgery (%)
Kim et al. (5)	20	20^*^	3 months^†^	PVA (20)	20 (100)	0 (0)	0 (0)	20 (100)	0 (0)^‡^	19 (95.0)	1 (5.0)	0 (0)
Ban et al. (9)	72	72^*^	6 months^†^	PVA (72)	27 (37.5)	45 (62.5)	27 (37.5)	0 (0)	0 (0)	NR	1 (1.4)	1 (1.4)
Waqas et al. (10)	8	8	3.3 months	Onyx (8)	8 (100)	0 (0)	6 (75)	2 (25.0)	0 (0)	8 (100)	0 (0)	0 (0)
Saitoh et al. (11)	8	8^*^	28.9 months	NBCA (7) NBCA+PVA (1)	1 (12.5)	7 (87.5)	0 (0)	8 (100)	0 (0)	NR	1 (12.5)	1 (12.5)
Okuma et al. (4)	17	17^*^	26.3 months	NBCA (11) Embosphere (3) both (3) coil (1)	2 (11.8)	15 (88.2)	2 (11.8)	11 (64.7)	0 (0)	NR	0 (0)	0 (0)
Nakagawa et al. (12)	20	20^*^	24 weeks^†^	NBCA (20)	0 (0)	20 (100)	0 (0)	20 (100)	0 (0)	NR	0 (0)	0 (0)
Link et al. (13)	49	60	>6 weeks	PVA (49)	50 (83.3)	10 (16.7)	42 (70.0)	8 (13.3)	0 (0)	41 (91.1)	4 (8.9)	4 (8.9)
Yajima et al. (14)	18	18^*^	18.1 months	NBCA (18)	2 (11.1)	16 (88.9)	2 (11.1)	15 (83.3)	0 (0)	NR	0 (0)	0 (0)
Shotar et al. (15)	89	89^*^	3 months^†^	Microspheres with or without coil (81) coil (5) NBCA (5)	0 (0)	89 (100)	0 (0)	22 (21.2)	6 (6.6)	NR	7 (7.7)	4 (2.2)
Rajah et al. (16)	46	46^*^	8 weeks	Onyx (43) NBCA (1)	42 (91.3)	4 (8.7)	37 (80.4)	5 (10.9)	0 (0)	38 (86.5)	5 (11.4)	5 (11.4)
Ng et al. (17)	19	22	3 months	PVA and/or coil (21)	0 (0)	22 (100)	0 (0)	0 (0)	0 (0)	NR	1 (4.5)	1 (4.5)
Mureb et al. (18)	8	8	89 days	PVA (8)	8 (100)	0 (0)	8 (100)	0 (0)	0 (0)	8 (100)	0 (0)	0 (0)
Joyce et al. (19)	121	151	90 days†	Coil (6) liquid (30) particles (38) liquid + coil (2) particles + coil (72) particles + liquid (1)	134 (88.7)	17 (11.3)	79 (52.3)	55 (36.4)	3 (2.0)	130 (94.2)	9 (7.4)	9 (7.4)
Fan et al. (20)	7	7	4–6 months	Absolute alcohol (7)	0 (0)	7 (100)	0 (0)	NR	0 (0)	7 (100)	0 (0)	0 (0)
Wei et al. (21)	10	20	112 days	Coil (10)	0 (0)	20 (100)	0 (0)	0 (0)	0 (0)	NR	0 (0)	0 (0)
Tiwari et al. (22)	10	13	160 days	Embospheres and/or coil/gel-form (8)	13 (100)	0 (0)	7 (53.8)	6 (46.2)	0 (0)	12 (92.3)	0 (0)	0 (0)
Tanoue et al. (23)	15	15	28 days^†^	NBCA (13) Embosphere (2)	15 (100)	0 (0)	15 (100)	NR	0 (0)	NR	3 (20.0)	3 (20.0)
Schwarz et al. (24)	41	44	321 days	PVA (44)	0 (0)	44 (100)	0 (0)	NR	0 (0)	40 (90.9)	2 (4.5)	2 (4.5)
Petrov et al. (25)	10	15	111 days	Squid (15)	15 (100)	0 (0)	9(60)	6 (40.0)	0 (0)	10 (100)	0 (0)	0 (0)
Lee et al. (26)	22	31	>2 weeks	Liquid (11) PVA (9) PVA and coil (11)	31 (100)	0 (0)	28 (90.3)	3 (13.6)	1 (3.2)	15 (48.4)	2 (6.5)	2 (6.5)
Kan et al. (27)	138	138^*^	94.9 days	PVA + coil (70) PVA (38) liquid (37) coil (5) liquid + coil (2)	138 (100)	0 (0)	92 (66.7)	42 (33.3)	3 (2.2)	134 (87.0)	10 (7.2)	9 (6.5)
Gomez-Paz et al. (28)	23	27	3 months	PVA + coil (27)	27 (100)	0 (0)	27 (100)	0 (0)	0 (0)	27 (100)	1 (3.7)	3 (11.1)
Scovile et al. (29)	208	208	6 months^†^	Particles; PVA, Embosphere (154) liquid; NBCA, Onyx (54)	192 (92.1)	16 (7.9)	133 (63.9)	59 (28.3)	11 (5.3)^‡^	126 (60.6)	NR	10 (4.8)
Saway et al. (30)	100	100^*^	1.9 months	Onyx (29) Particles (58) particle and coil (13)	0 (0)	100 (100)	0 (0)	10 (10.0)	1 (1.0)	100 (100)	2 (2.0)	2 (2.0)
Samarage et al. (31)	37	37^*^	NR	NBCA (38) PVA (9) Onyx (3) combination (17)^*^	19 (51.4)	18 (49)	19 (51.4)	NR	3 (8.1)	18 (48.6)	5 (13.5)	5 (13.5)
Salie et al. (32)	52	52	100 days	Particle coil Liquid combination (NR)	0 (0)	52 (100)	0 (0)	NR	NR	47 (90.4)	3 (5.8)	2 (3.8)
Onyinzo et al. (33)	50	50	3.4 months	PVA and/or coil (50)	19 (38.0)	31 (62.0)	19 (38.0)	NR	0 (0)	13 (59.0)	1 (4.5)	1 (4.5)
Mir et al. (34)	56	56^*^	90 days	PVA and/or coil (NA) Onyx (NA) NBCA (NR)	56 (100)	0 (0)	35 (62.5)	21 (37.5)	2 (3.6)	35 (62.5)	1 (1.8)	1 (1.8)
Magidi et al. (35)	61	61^*^	3 months^†^	NBCA (61)	61 (100)	0 (0)	31 (50.8)	30 (49.2)	2 (3.3)	59 (96.7)	3 (4.9)	2 (3.3)
Khorasanizadeh et al. (36)	78	94	114 days	PVA and coil (82) coil (12)	80 (85.1)	14 (14.9)	72 (76.6)	8 (8.5)	2 (2.1)	67 (78.8)	8 (8.5)	8 (8.5)
Housley et al. (37)	44	48	12–60 weeks	Onyx (48)	48 (100)	0 (0)	48 (100)	0 (0)	NR	38 (79.2)	2 (4.2)	2 (4.2)
Fuentes et al. (38)	322	322	5 years	NR	322 (100)	0 (0)	286 (88.8)	36 (11.2)	NR	NR	NR	55 (17.1)
Enriquiz-Marulanda et al. (39)	36	45	72 days	PVA + coil (43) coil (2)	35 (77.7)	10 (22.2)	35 (77.7)	NR	1 (2.2)	28 (71.8)	1 (2.6)	5 (11.1)
Dofuku et al. (40)	9	9	103 days	NBCA (9)	0 (0)	9(100)	0 (0)	9 (100)	0 (0)	NR	0 (0)	0 (0)
Catapano et al. (41)	66	84	180 days†	Onyx (66) PVA or coil or both (13) NBCA (5)	53 (63.1)	31 (36.9)	53 (63.1)	NR	1 (1.2)	67 (91.8)	3 (3.6)	3 (3.6)
Carpenter et al. (42)	23	23^*^	4.1 months	PVA (23)	0 (0)	23 (100)	0 (0)	NR	6 (26.0)	NR	2 (9.1)	2 (9.1)
Wali et al. (43)	8	8	>3 months	PVA and helical coil (8)	7 (87.5)	1 (12.5)	7 (87.5)	0 (0)	0 (0)	8 (100)	0 (0)	0 (0)
Shehabeidin et al. (44)	97	97	4.2 months (Onyx) 3.0 months (PVA)	Onyx (49) PVA (48)	48 (53.3)	NR	48 (53.3)	NR	0 (0)	NR	18 (18.6)	13 (13.4)
Seok et al. (45)	9	13	4.7 months	PVA and Gelform (8)	8 (61.5)	5 (38.5)	8 (61.5)	4 (30.8)	0 (0)	12 (92.3)	1 (7.7)	1 (7.7)
Salem et al. (46)	530	636	121 days	coil and particles (248) liquid (228)	468 (74.3)	162 (25.8)	318 (50.4)	150 (23.9)	16 (3.0) ‡	490 (87.5)	36 (6.8)	36 (6.8)
Martinez-Gutierrez et al. (47)	57	66	20 days	Particles (NR) coil (NR) Onyx (NR)	66 (100)	0 (0)	25 (37.9)	41 (62.1)	NR	NR	11 (16.7)	4 (6.1)
Liu et al. (48)	53	53	6 months^†^	PVA and NBCA (53)	31 (58.5)	22 (41.5)	31 (58.5)	NR	NR	48 (90.6)	1 (2.4)	0 (0)
Krothapalli et al. (49)	116	116	29 days (liquid) 35 days (particles)	NBCA (48) PVA (68)	68 (58.6)	48 (41.4)	68 (58.6)	NR	1 (0.9)	NR	6 (5.2)	2 (1.7)
	2,783	3,027			2,114 (69.8)	907 (31.0)	1,617 (53.4)	591 (24.6)	58 (2.4)	1,645 (83.8)	155 (6.2)	197 (6.4)

NR, not reported; MMAE, middle meningeal artery embolization; cSDH, chronic subdural hematoma; PVA, polyvinyl alcohol; NBCA, N-butyl cyanoacrylate. ^*^Treatment of bilateral cSDH counted as one treatment. ^†^Follow-up period was set as the evaluation period in the study. ^‡^Complications with unknown details or unknown association with MMAE were not counted.

MMAE was performed as the sole treatment in 69.1% of patients and prophylactically after or before surgical evacuation in 30.7%. The details in six (0.2%) patients were not reported.

Among all cases, 23.7% were recurrent cSDH after previous surgical evacuation, and 52.7% were treated as upfront MMAE.

The embolic agents included particles, liquid agents, and coils and were used alone or in combination. Details are shown in Table 1. The mean follow-up duration ranged from 20 days to 5 years.

Following MMAE, the rates of cSDH resolution, recurrence, and surgical rescue at the last follow-up were 83.8%, 6.2%, and 6.4%, respectively.

In most studies, no serious complications occurred after MMAE. The rate of severe complications associated with MMAE was 1.0% (28 of 2,783 patients), including 10 cases of cerebral infarction, 5 cases of visual loss, 4 cases of facial palsy, 2 cases of cerebral hemorrhage, 3 cases of MMA arteriovenous fistula, 1 case of MMA rupture, 1 case of aortic dissection, 1 case of femoral artery occlusion, and 1 case of catheter entrapment. The rate of overall complications associated with MMAE was 2.3% (58 of 2,783 patients), including seizures, headache, renal dysfunction, transient neurological symptoms (diplopia and aphasia), and puncture site hematoma. Two overall treatment-related mortalities due to cerebral hemorrhage and femoral artery occlusion were observed.

### Rescue surgery rate in MMAE-alone treatment group and prophylactic MMAE group

The hematoma resolution, recurrence, and retreatment rates in the MMAE-alone treatment group (*n* = 815) were 86.7%, 6.3%, and 9.6%, respectively, whereas those in the prophylactic MMAE with combined surgery group (*n* = 370) were 95.6%, 4.4%, and 3.4%, respectively. The percentage of symptomatic patients in the MMAE-alone group and prophylactic MMAE group was 94.0% and 97.7%, respectively. These results are shown in Table 3.

**Table 3:** Comparison between reports of MMAE-alone treatment and reports of prophylactic MMAE treatment.

	MMAE alone	Prophylactic MMAE
Total no of patients	797	370
Total no of embolization	815	386
Age (mean)	68.8	74.6
Antithrombotic therapy (%)	271(34.3)	229 (64.9)
Symptomatic cSDH (%)	109 (94.0)	256 (97.7)
Recurrent cSDH (%)	205 (25.9)	61 (35.9)
Resolution (%)	353 (86.7)	194 (95.6)
Recurrence (%)	31 (6.3)	17 (4.4)
Rescue surgery (%)	78 (9.6)	13 (3.4)

MMAE, middle meningeal artery embolization; cSDH, chronic subdural hematoma.

## Discussion

The standard treatment for cSDH is still surgical treatment, however cSDH has a high recurrence rate (10%–20%) after a single surgical evacuation (2, 3).

The medical treatment options for cSDH have been extensively investigated, these studies had no remarkable effectiveness in preventing recurrence of cSDH (2, 3, 50–52) and an optimal treatment strategy for preventing cSDH recurrence has not yet been established.

The pathophysiology of cSDH involves formation of inflammatory membranes and self-sustaining neoangiogenesis and fibrinolysis, leading to a high prevalence of rebleeding from fragile capillaries (6, 7). These vessels are derived from the dura matter and perfused mainly by the distal branches of the MMA; therefore, MMAE could be an interesting paradigm for the treatment of cSDH.

During the past few years, the number of reports on the efficacy of MMAE has rapidly increased. In the present systematic review, the cSDH resolution, recurrence, and rescue surgical treatment rates at the last follow-up after MMAE in all patients were 83.8%, 6.2%, and 6.4%, respectively.

MMAE can be divided into two main categories according to the purpose of treatment: curative MMAE with the expectation of hematoma reduction to avoid surgical intervention (or in place of surgical hematoma removal) and prophylactic MMAE as a preventive treatment for recurrence in combination with hematoma evacuation.

### MMAE as a sole treatment for cSDH

The three initially reported indications for MMAE alone were failure of conservative treatment or asymptomatic or mildly symptomatic patients (to avoid surgery), advanced age and use of antiplatelet or anticoagulant drugs (as an alternative treatment considering the invasiveness of surgery), and prevention of recurrence in patients with recurrent disease after surgical treatment (53).

In an evaluation of the efficacy of MMAE alone, Housley et al. (37) compared the outcomes of 48 propensity-matched patients with cSDH who underwent either surgery alone or MMAE alone as initial treatment. There was a significant hematoma reduction in the surgery group immediately after surgery; after 12 weeks of treatment, however, there was no significant difference in hematoma reduction between the two groups. Furthermore, the recurrence rate was significantly lower in the MMAE group (22.9% vs. 4.2%) (37). Kim (5) compared the outcomes of 23 patients who received conventional treatment and 20 patients who received MMAE among 43 patients who developed recurrent cSDH after surgical treatment. The MMAE group showed better prevention of recurrence and earlier brain re-expansion despite the fact that patients of advanced age were significantly more likely to use antithrombotic drugs in the MMAE group (5). These studies show that the effect of MMAE alone for reducing a hematoma is equivalent to evacuation surgery alone in long-term follow-up, and the effect of preventing recurrence may surpass the effect of evacuation surgery alone.

Some studies showed a good hematoma reduction effect of MMAE alone, even for massive cSDH. Gomez-Paz et al. (28) reported that patients with massive cSDH with a midline shift of ≥5 mm were treated with upfront MMAE and showed good improvement in symptoms and imaging findings under careful follow-up.

In the present review, surgical evacuation was needed in 9.6% of patients in the MMAE-alone group, suggesting that some patients developed hematoma enlargement or neurological deterioration even after MMAE. Therefore, careful follow-up is important, especially in the early postoperative period after patients undergo MMAE alone.

Some reports also suggested that MMAE alone is superior to surgery alone in terms of cost-effectiveness because of the reduced need for additional therapeutic intervention in the treatment of cSDH (54, 55).

### MMAE combined with evacuation for cSDH

In this study, prophylactic MMAE showed a favorable hematoma reduction rate, recurrence rate, and reoperation rate. The major advantage of MMAE is its effect in preventing recurrence. In this review, the reoperation rate was 1.4% to 4.9% in the MMAE with surgical treatment group and 11.6% to 18.8% in the conventional surgical treatment group (9, 15, 32, 33).

Okuma et al. (4) reported the following predictive factors for refractory cSDH after burr-hole surgery: use of antiplatelet drugs or anticoagulants, blood coagulation disorder, hepatic dysfunction, hemodialysis, terminal malignancy, advanced age (>80 years), cerebral atrophy, large preoperative hematoma volume (>150 mL), niveau formation, post cerebrospinal fluid shunt placement, no placement of a drain during surgery, postoperative residual air (>20%), and multiple recurrences. The authors also stated that such predictive factors have the potential to be good indications for prophylactic MMAE.

The selection of appropriate cases with respect to the indication for prophylactic MMAE is important from the standpoint of medical economics. Further investigation of the indications for prophylactic MMAE is warranted.

### Complications of MMAE

The rate of MMAE-related complications in this review was 2.3%. Although serious complications such as cerebral infarction, visual loss, and facial palsy rarely occurred, their development suggests the possibility of stray embolic material entering high-risk anastomosis sites. Because of the potential for anastomosis in MMAs that involve the retinal artery and vasa nervorum of the facial nerve, embolization can lead to serious and permanent complications such as blindness and facial nerve palsy (29). During the embolization procedure, the microcatheter should be positioned distal to the branch, and attention should be paid to the findings of reflux during embolic agent injection (44). In addition, in patients with a high risk of migration of embolic material to a compromised anastomosis, it is important to perform a provocation test using lidocaine and abort the embolization based on the result of this test, if necessary (16). Although MMAE is a safe and easy procedure, close attention is needed when working with high-risk anastomoses to prevent complications during MMAE.

### Factors that predict a good outcome after MMAE

With the increase in reports of MMAE for treatment of cSDH, there has also been an increase in reports regarding factors that affect the efficacy of MMAE. Salem et al. (46) evaluated various factors in 530 patients treated with MMAE; they found that an MMA main trunk diameter of <1.5 mm and anticoagulant medication use were factors associated with higher retreatment rates and that the use of liquid embolic material was a factor associated with lower retreatment rates. Some reports have suggested that the use of liquid embolic material is as effective and safe as the use of particles (29, 44, 49).

In addition, distal (midline) penetration of the embolizing material has been cited as a factor that shortens the time to hematoma clearance (31, 41). Among all anticoagulants, only factor Xa inhibitors were reported to be associated with retreatment (38).

### Limitations

This review had three main limitations. First, most of the reports were retrospective, and they contained various indications for MMAE and definitions of resolution. Thus, the statistical examination was limited, leaving potential for important selection bias. Second, the content of the articles was mixed, with some studies limited to MMAE (whether upfront MMAE, prophylactic MMAE, or MMAE for recurrence after surgery), others comparing MMAE with surgery, and still others focusing on the clinical course or radiographic findings. Third, some studies lacked information on the timing of MMAE and surgical treatment and did not clearly indicate whether complications in cases of combined surgery and MMAE were caused by surgery or MMAE. These factors made statistical evaluation difficult in this review. Further prospective randomized trials are required to establish the clear indications for MMAE as an initial treatment or combined treatment with evacuation surgeries.

## Conclusion

This study shows that MMAE alone is, although not immediate, as effective as evacuation surgery alone in reducing hematoma. The study also shows that combined treatment has a lower recurrence rate than evacuation surgery alone. Because MMAE is a safe procedure, it should be considered for patients with cSDH, especially those with a high risk of recurrence.

## Data availability statement

The original contributions presented in the study are included in the article/supplementary material, further inquiries can be directed to the corresponding author.

## Author contributions

YO: Conceptualization, Data curation, Formal analysis, Investigation, Writing – original draft. TI: Supervision, Writing – review & editing.
